# Distributed Formation Control of Multi-Robot Systems with Path Navigation via Complex Laplacian

**DOI:** 10.3390/e25111536

**Published:** 2023-11-11

**Authors:** Xiru Wu, Rili Wu, Yuchong Zhang, Jiansheng Peng

**Affiliations:** 1 School of Electronic Engineering and Automation, Guilin University of Electronic Technology, Guilin 541004, China; xiruwu@guet.edu.cn (X.W.); yczhang_1128@163.com (Y.Z.); 2 Key Laboratory of AI and Information Processing, Education Department of Guangxi Zhuang Autonomous Region, Hechi University, Hechi 546300, China; pengjs@hcnu.edu.cn

**Keywords:** complex Laplacian, formation control, path navigation, multi-robot systems

## Abstract

This paper focuses on the formation control of multi-robot systems with leader–follower network structure in directed topology to guide a system composed of multiple mobile robot agents to achieve global path navigation with a desired formation. A distributed linear formation control strategy based on the complex Laplacian matrix is employed, which enables the robot agents to converge into a similar formation of the desired formation, and the size and orientation of the formation are determined by the positions of two leaders. Additionally, in order to ensure that all robot agents in the formation move at a common velocity, the distributed control approach also includes a velocity consensus component. Based on the realization of similar formation control of a multi-robot system, the path navigation algorithm is combined with it to realize the global navigation of the system as a whole. Furthermore, a controller enabling the scalability of the formation size is introduced to enhance the overall maneuverability of the system in specific scenarios like narrow corridors. The simulation results demonstrate the feasibility of the proposed approach.

## 1. Introduction

In recent years, due to the development of artificial intelligence, robotics, sensors, and communication technologies, team-working robots have gradually replaced complex individual robots in both civilian and military applications. Such multi-robotic systems can help humans tackle challenges that involve vast, inaccessible, and dangerous areas [1,2,3,4,5]. In robotics, a common need is for groups to behave in ways that not only demonstrate shape but also move in a coordinated manner. In this regard, the formation control algorithm provides a certain solution according to the sensing capability of the agent and the required geometric pattern [6].

Formation control in multi-robot systems utilizes local interactions among robot agents to achieve formation group behavior in multi-robot systems, where each individual robot typically moves towards specific targets or directions in a certain geometric shape. The desired geometric shape can be characterized in several ways, such as the desired distances between robot pairs [7,8], the desired distances and angles between robot pairs [9,10], or the relative position of a robot agent to its neighboring robot agent [11,12]. Due to the wide-ranging applications of formation control in various fields, such as target encirclement [13], payload transportation [14], and environmental monitoring [15], scientists have developed a series of reliable methods for controlling and coordinating robot crowds or general multi-agent systems [16,17]. But at present, there is still a lot of research space in the dynamic formation and formation change of multi-robot.

Existing typical methods for formation control include leader–follower approaches [18,19], behavior-based approaches [20], virtual structure approaches [21], and graph theory-based approaches [22]. Nowadays, these research methods have become increasingly intertwined and difficult to strictly distinguish. Particularly, graph theory-based approaches have gained widespread attention from researchers in recent years due to their ability to leverage mature graph theory knowledge for the design of formation control laws, formation configuration, and information flow within the formation.

Control techniques using the complex Laplacian matrix, among various research approaches in graph theory, have been applied to solve formation control problems [23,24,25,26,27]. Formation using a real Laplacian matrix in the graph theory method is to set certain deviations for each agent on the basis of consistency protocols so that each agent finally forms a preset formation. Like most methods of formation at present, once the shape of the team is completed, switching to another formation often requires changes to the control protocols of many or even all of the agents.

The formation control based on a complex Laplacian matrix solves this problem to some extent. When the switched formation is the combination of translation, rotation, and contraction of the original formation, the relative position of some agents (double leaders) is changed by using the complex Laplacian matrix to reach the specified target, and the control protocol of other agents remains unchanged. According to the updated control protocol, the system can naturally switch to the new formation with the combination of translation, rotation, and contraction of the original formation.

A similar formation based on the complex Laplacian matrix introduced by [28], which is a sufficient and necessary condition that can be realized in the plane, is that the communication graph is two-rooted. The formation of complex Laplace in an undirected graph has been discussed by Lin et al. [23], in which a new distributed control protocol based on complex Laplacian is introduced. In addition to this, the methods based on the complex Laplacian matrix have been proposed in [24,25,26,27] to deal with control protocols in leader–follower communication networks. The authors in [27] introduce their control law to achieve dynamic formation of the system and extend it to achieve mobile formations where the leader tracks time-varying reference velocities. In this control law, besides the given reference velocity information, all individual robot agents need to know their own velocity information. In order to reduce the consumption of inter-agent communication, some scholars also try to introduce event-driven algorithms into complex Laplacian matrix formation control, as shown in the literature [29,30,31]. However, the formation control technique based on the complex Laplacian matrix proposed in the above technique simply realizes the desired formation control of the whole formation, which is far from enough to extend its practical application.

Compared with the techniques mentioned above, this paper proposes a multi-robot distributed formation control strategy based on a complex Laplacian matrix, and combines the path navigation algorithm with similar dynamic moving formation control to realize the global formation navigation of the whole system. In summary, the main contribution of this paper lies in the following aspects:

(1) This paper proposes a distributed formation control law based on the complex Laplacian matrix, which can enable a group of mobile robots to achieve the desired formation at a specified speed and control the realization of similar formation of multiple robot systems using the relative positions of the two leaders.
(2) Considering the application extension of the multi-robot system under the requirements of specific task scenarios, and based on the successful achievement of similar formation control, the whole multi-robot system is placed in the preset scene map, and the corresponding path navigation algorithm is adopted for the leader to realize the global navigation application of the system. The whole multi-robot system can reach the designated end point from the desired formation without collision.
(3) Based on the current formation change control requirements, the processing capability of multi-robot systems for special scenes such as narrow and long corridors is increased. Additionally, it also leverages the scalability formation function inherent in similar formations to improve the basic control rules. This refinement facilitates the overall scaling of formation by adjusting the anticipated spacing between the two leaders, thereby enhancing accessibility in specific scenarios. The combination of this feature with a global navigation application will help to further enhance the navigation control strategy of multi-robot systems.

The structure of this paper is as follows. Section 2 provides an introduction to the necessary background knowledge and terminology. Section 3 presents the distributed formation navigation control strategy based on the complex Laplacian matrix, which is divided into three parts: similar formation, global navigation, and narrow lane traffic. Simulation results are presented in Section 4, and finally, Section 5 concludes the paper.

*Notation:* In this section, we will introduce the basic concepts and terms that will be used in this article. The symbol ℂ represents a set of complex numbers, whereas the symbol $N^{+}$ represents a set of positive integers. For the complex number w=a+bj, its real and imaginary parts are represented by Re(w) and Im(w), respectively. The symbol $1_{n}$ represents an n-dimensional vector, and the symbol $I_{n}$ represents an n-by-n identity matrix.

## 2. Preliminaries and Terminology

### 2.1. Graph Theory

In a multi-robot system, its communication topology can be described using a directed graph G={V,E}. The non-empty set $V=\{v_{1},v_{2},v_{3}\cdots ,v_{n}\}$ represents a set of individual robot nodes, whereas the other non-empty set E⊆V×V is a set of node pairs used to represent the information links between two robot nodes, which represent edges. For any node pair $\left(v_{j},v_{i}\right)$ belonging to the non-empty node set V, if $(v_{j},v_{i})\in E$ holds, the edge is then considered as a directed line segment from node $v_{j}$ to node $v_{i}$, where node $v_{j}$ is the in-neighbor of node $v_{i}$, and node $v_{i}$ is the out-neighbor of node $v_{j}$. We define the in-neighbor set of node $v_{i}$ as $N_{i}=\{v_{j}:(v_{j},v_{i})\in E\}$.

Here are two related concepts from [25].

**Definition** **1.** *In a directed graph G, for two paths from subset U⊂V* *to node v, if there are no common nodes except for node v, then these two paths are considered non-intersecting. Furthermore, if there exists a path from subset U* *to node v, then v* *is said to be reachable from U. If, after removing any node other than node v, there still exists a path from subset U* *to node v, then node v* *is said to be 2-reachable from the non-singleton subset U*

**Definition** **2.** *For a directed graph G, if there exists a non-singleton subset U* *consisting of two nodes, it is 2-reachable from these two nodes to any other node in U, then the directed graph G* *is called a two-rooted graph, and these two nodes are referred to as roots in the directed graph G.*

**Figure 1:**
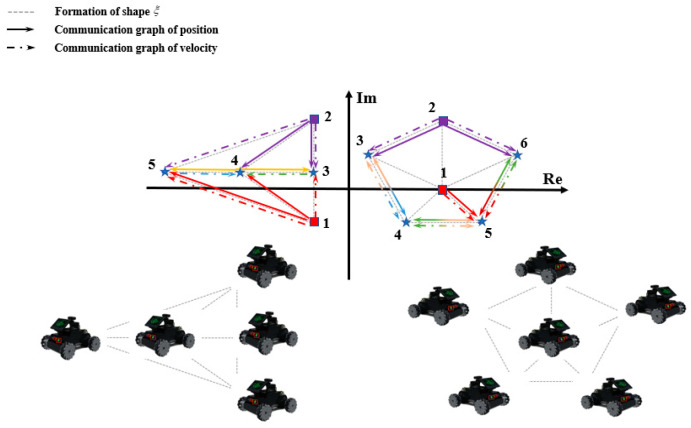
Multi-robot system communication topology graph.

In Figure 1, there are two specific examples illustrating the communication topology of two multi-robot systems. The solid arrows represent position information communication graphs, the dashed arrows represent velocity information communication graphs, and each arrow color represents its source node color. Firstly, in a five-agent system, if set U contains nodes 1 and 2, then any other individual node excluding nodes 1 and 2 is selected. Taking node 5 as an example, it is not difficult to observe that, in both the position information communication graph and the velocity information communication graph, even when nodes 3 or 4 are removed, there still exists a reachable path from set U={1,2} to node 5. Therefore, according to Definition 1, node 5 is 2-reachable for set U in both communication graphs. Similarly, node 3 and node 4 are also 2-reachable for set U. In accordance with Definition 2, both the communication graphs of the five-agent system in Figure 1 have two roots, where nodes 1 and 2 are the two roots of the graph. Furthermore, since the two communication graph topologies of the six-agent system are identical, it can be easily verified if setting U as {1,2} is for the six-agent system, and it can also be deduced if the two communication topology graphs of the system have two roots.

Finally, a complex Laplacian matrix L is introduced for the directed graph G. Each edge $\left(v_{j},v_{i}\right)$ in the graph is associated with a complex-valued number $w_{ij}$ in the matrix. Its specific definition is as follows: for the off-diagonal entries in the matrix, if there is an $j\in N_{i}$, its value is $-w_{ij}$, otherwise it is 0. For the diagonal entries, their values are $\sum_{j\in N_{i}}w_{ij}$. Its formulaic representation is provided as follows:

(1) $$L(i,j)=\left\{ \begin{array}{l} -w_{ij}\;j\in N_{i}\;and\;i\neq j\;\\ 0\;j\notin N_{i}\;and\;i\neq j\\ \sum_{j\in N_{i}}w_{ij}\;others \end{array}\right.\;(w_{ij}\neq 0).$$

The complex Laplacian matrix L differs from the familiar real Laplacian matrix in that the weights $w_{ij}$ in the matrix are complex-valued. When all the values in $w_{ij}$ are real numbers, L becomes a real Laplacian matrix. Therefore, the complex Laplacian matrix also possesses some properties that are similar to the real Laplacian matrix. For example, the sum of rows in the real Laplacian matrix is zero, and this property is also reflected in the complex Laplacian matrix.

**Remark** **1.** *Control techniques using the complex Laplacian matrix, among various research approaches in graph theory, have been applied to solve formation control problems. Main features of this approach can be listed as the following: (i) four degrees of freedom, two for translation, one for each of rotation and scaling, are allowed in this approach; (ii) a common coordinate system is no longer required as opposed to consensus-based formation control schemes; and (iii) unlike distance-based formation control, the complex Laplacian-based approach leads to linear control laws, meaning that the global asymptotic stability of the algorithm can be guaranteed [26].*

### 2.2. Multi-Bobot Two-Dimensional Geometric Similar Formation

Several concepts related to two-dimensional geometry formation are presented next. Geometric formation in a two-dimensional plane is essentially defined as an ordered set of points in the plane. In this paper, we define a specific coordinate system in the two-dimensional plane. In this coordinate system, a complex number can determine a specific point, where the real part represents the x-coordinate and the imaginary part represents the y-coordinate. Next, a vector $\xi ={\left[\xi_{1},\xi_{2},\xi_{3}\cdots ,\xi_{n}\right]}^{T}$ consisting of n complex numbers is designed, where each complex number represents a robot agent node. The position of each robot agent can be uniquely determined by the complex number in vector ξ. Therefore, it can fully characterize the relative positional relationships between robot agent nodes using only vector ξ, which is also referred to as the basic formation. Additionally, a similar formation set for vector ξ is defined:

(2) $$S_{\xi }=\{c_{1}1_{n}+c_{2}\xi \}$$

In the remainder of this paper, we will name all the elements in $S_{\xi }$ as similar formations of ξ. The so-called similar formation refers to the formation shape formed based on the basic formation ξ through a combination of translation, rotation, and scaling. Among them, $c_{1}$ can carry out any two-dimensional translation of ξ, whereas $c_{2}$ makes possible arbitrary rotation and scaling, and $c_{1},c_{2}\in \mathbb{C}$. By adjusting the weights of $c_{1}$ and $c_{2}$, it is possible to represent similar formations in basic formation ξ. More intuitively, $c_{2}$ can also be expressed as $c_{2}=\kappa e^{\theta j}$, where κ represents the scaling factor and θ represents the rotation angle. According to [25,26], it can be concluded that if there exists an edge $\left(v_{j},v_{i}\right)$ with a complex number weight $w_{ij}$ in the directed graph G such that the null space of L satisfies $ker(L)=S_{\xi }$, then a similar formation of ξ can be achieved.

Equation (2) implies that the complex Laplacian matrix L has only two zero eigenvalues corresponding to two linearly independent eigenvectors $1_{n}$ and ξ. In order for $ker(L)=S_{\xi }$ to hold, there needs to be

(3) $$L1_{n}=0,\;L\xi =0,\;rank(L)=n-2.$$

The complex Laplacian matrix possesses the same property as the real Laplacian matrix, which is the property of having a zero row sum, i.e., $L1_{n}=0$. Therefore, it can be further deduced that in a specific topology of a directed graph G, for any L and basic formation ξ, Lξ=0 can be achieved by selecting complex weight values $w_{ij}$. Another formula representation of this is as follows:

(4) $$\sum_{j\in N_{i}}w_{ij}(\xi_{j}-\xi_{i})=0,\;i=1,2,3,\cdots ,n$$

The complex weights $w_{ij}$ in the linear constraint (4) can be designed in a distributed manner to encode the desired formation information using complex weights $w_{ij}$, and by utilizing the formation base vector ξ and linear constraint (4), appropriate complex weights can be designed. Specific design methods are given in reference [23]. Typically, the solution for $w_{ij}$ is not unique, and the agent i can choose a solution arbitrarily.

Moreover, it is indicated in [28] that as long as the directed graph G satisfies Lemma 1, rang(L)=n−2 is almost certain to hold for any weights satisfying Equation (3).

**Lemma** **1.** *For a general ξ∈ℂ* *and when any two components in ξ* *are not identical to each other. A sufficient necessary condition for a similar formation of shape ξ* *is realizable in a directed graph G* *is only when the directed graph G* *is two-rooted.*

It is worth mentioning that Equation (3) represents a necessary algebraic condition for achieving similar formations of multi-robot two-dimensional plane with respect to ξ, whereas Lemma 1 provides a graph theory condition.

## 3. Formation Navigation Control via Complex Laplacian

### 3.1. Problem Statement

In a two-dimensional plane, consider a multi-robot system with n individual agents, and label them as 1,2,3,⋯n. Among them, agents 1 and 2 are designated as co-leaders of the system. The leaders do not receive information transmission from other individual agents, including each other. Therefore, the Laplacian matrix form for a directed graph G satisfying Lemma 1 in the general sense is as follows:

(5) $$L=\left[\begin{array}{cc} 0_{2\times 2} & 0_{2\times (n-2)}\\ L_{lf} & L_{ff} \end{array}\right].$$

Furthermore, in order to achieve coordinated motion of the multi-robot system, it is necessary to ensure the consistency of individual velocities. Therefore, this paper constructs another directed graph $G_{v}$ for the same multi-robot system, which is used to transmit velocity information among the individual agents. To differentiate it from the aforementioned directed graph G, they will be referred to as the position information communication graph G and the velocity information communication graph $G_{v}$ based on the type of information it transmits. The velocity information communication graph $G_{v}$ differs from the position information communication graph G in that the directed edges between nodes in $G_{v}$ represent the transmission of velocity information between individuals, but it still has similar properties to the graph G. Without loss of generality, graph $G_{v}$ may have a different topology from the directed graph G. By attaching a real-valued weight to the directed edges in graph $G_{v}$, its real Laplacian matrix $H=\left[h_{ij}\right]$ is as follows:

(6) $$h_{ij}=\left\{ \begin{array}{l} -h_{ij}\;j\in N_{i}(G_{v})\;and\;i\neq j\;\\ 0\;j\notin N_{i}(G_{v})\;and\;i\neq j\\ \sum_{j\in N_{i}(G_{v})}h_{ij}\;others \end{array}\right.$$

where $N_{i}(G_{v})$ represents the set of in-neighbors of node $v_{i}$ in the velocity communication graph $G_{v}$, and $h_{ij}$ represents the real-valued weight on the edges $\left(v_{j},v_{i}\right)$ in the velocity communication graph $G_{v}$. 

Similarly, in the velocity communication graph $G_{v}$, the leader node has no incoming edges, which means the leader does not receive velocity information from other agents, including the other leader. Likewise, a Laplacian matrix H with velocity information transfer similar to Equation (5) can be obtained as follows:

(7) $$H=\left[\begin{array}{cc} 0_{2\times 2} & 0_{2\times (n-2)}\\ H_{lf} & H_{ff} \end{array}\right].$$

In our assumption, we consider no information exchange between the two leaders. Therefore, the elements in the first two rows of matrices L and H are both zero. It should be noted that the control method to be proposed next is also applicable when there is an information exchange between the leaders. In this case, matrices L and H will not have the same structure as Equations (1) and (6). Non-zero elements may appear in the first two rows of the matrices, but under the same assumption mentioned above, similar formations with respect to the basic formation ξ can still be achieved.

In the two-dimensional plane, for a multi-robot system consisting of *n* individual agents, a control strategy is designed based on the given position communication graph G and velocity communication graph $G_{v}$ in a dual-leader network structure, which can achieve stable formation and speed synchronization of the multi-robot system. Furthermore, it ensures that the system can reach the destination without collision in a given map environment by utilizing the transformations of similar formation and a specific path navigation algorithm. 

### 3.2. Similar Formation Control

In this section, we assume a robot system in which both the position communication graph G and velocity communication graph $G_{v}$ satisfy Lemma 1. Also, the positions of n individuals in the system are represented by a set of complex vectors $p={\left[p_{1},p_{2},p_{3},\cdots p_{n}\right]}^{T}$, and the states of each individual agent in the system are modeled as the following first-order kinematic model:

(8) $${\dot{p}}_{i}=u_{i}\;i=1,2,3,\cdots ,n$$

Furthermore, we consider individuals 1 and 2 as co-leaders of the system and design different controllers for the leaders and the followers:

(9) $$\left\{ \begin{array}{l} {\dot{p}}_{i}=\gamma_{i}\;i=1,2\\ {\dot{p}}_{i}=\gamma_{i}+d_{i}\sum_{j\in N_{i}}w_{ij}(p_{j}-p_{i})\;i=3,4,\cdots ,n \end{array}\right.$$

where $d_{i}$ is the system stability control parameter to be configured, $w_{ij}$ denotes the complex-valued weights on the edges $\left(v_{j},v_{i}\right)$ in the position communication graph G, and $\gamma_{i}$ represents the velocity control input. We assume that the two leaders travel at a uniform speed $V_{0}$, and through the velocity communication graph $G_{v}$, the dynamic velocity controller for the system can be expressed as:

(10) $$\left\{ \begin{array}{l} \gamma_{i}=V_{0}\;i=1,2\\ {\dot{\gamma }}_{i}=\sum_{j\in N_{i}(G_{v})}h_{ij}(\gamma_{j}-\gamma_{i})\;i=3,4,\cdots ,n \end{array}\right.$$

As mentioned above, matrix L is a complex Laplacian matrix associated with the location information communication graph G, whose elements are the complex weight of the corresponding directed edges in the graph. Therefore, its form is shown in Formula (1). Matrix H is a real Laplacian matrix associated with the velocity information communication graph $G_{v}$, whose elements are the real weight of the corresponding directed edges in the graph. Its form is similar to that of the complex Laplacian matrix L, as shown in Equation (6). Then, we set a stable matrix $D=diag(d_{1},d_{2},d_{3},\cdots ,d_{n})$ in order to give the matrix expression of the system equation of state:

(11) $$\left[\begin{array}{l} \dot{p}\\ \dot{\gamma } \end{array}\right]=\left[\begin{array}{cc} -DL & I_{n}\\ 0 & -H \end{array}\right]\left[\begin{array}{c} p\\ \gamma \end{array}\right].$$

As can be seen from Figure 1 of this paper, both the position information communication graph G and the velocity information communication graph $G_{v}$ constructed in this paper are double root graphs. Under this premise, based on the existing research on the multi-agent consistency problem based on the real Laplacian matrix, it is not difficult to know that in the velocity controller model $\dot{\gamma }=-H\gamma$ constructed based on the premise described in this paper, the feature root distribution is located on the right plane of the complex plane, so the velocity of the individual agents in the system can be stable and asymptotically consistent. In contrast, the eigenvalues of complex Laplacian matrices can be in either the left plane or the right half plane. Therefore, the stability control matrix parameter $d_{i}$ needs to be configured for system (9) to make the system asymptotically stable.

**Lemma** **2** [28]. *Given a two-root graph G* *and target formation $\xi ={\left[\xi_{1},\xi_{2},\xi_{3}\cdots ,\xi_{n}\right]}^{T}$, for almost all complex Laplacian matrices L* *satisfying formula (3), there exists a stable matrix D* *that can configure the eigenvalues of −DL* *to any desired position, except for two fixed zero eigenvalues.*

Based on Lemma 2, if a directed graph is a two-rooted graph, then there is always an invertible diagonal matrix D for almost all complex Laplacian matrices of the graph, which satisfies Formula (3), such that all eigenvalues of the matrix −DL are configured in the right half plane of the complex plane. Thus, we can say that under the distributed control strategy (9) and (10), n agents can asymptotically form a stable motion formation with the desired formation shape ξ and move at a preset speed, and the size of the formation depends on the initial positions ${\bar{p}}_{1}$ and ${\bar{p}}_{2}$ of the two leaders, which are also reflected in the following sections. In addition, an algorithm for finding a suitable complex control parameter $d_{i}$ is given in [28]. Therefore, the stability control parameter $d_{i}$ mentioned in Formula (9) can be found under the condition that Lemma 1 is satisfied.

Since this paper adopts a formation control method based on the complex Laplacian matrix, the element $d_{i}$ contained in a stable matrix D will also have complex values. Meanwhile, since there is no information exchange between the two leaders in the system, the first two rows of elements in L and H are zero. According to the controller shown in Formula (9), the first two elements of the stable matrix D can be directly set to 1. It should be pointed out that in the case of communication between the leaders, the first two control parameters in the stability matrix D still need to be configured.

Obviously, the steady-state solution of system (11) satisfies the following form:

(12) $$\left\{ \begin{array}{l} p^{*}(t)=(c_{1}+V_{0}t)1_{n}+c_{2}\xi \\ \gamma^{*}(t)=V_{0}1_{n} \end{array}\right.$$

where

(13) $$\left[\begin{array}{l} c_{1}\\ c_{2} \end{array}\right]={\left[\begin{array}{cc} 1 & \xi_{1}\\ 1 & \xi_{2} \end{array}\right]}^{-1}\left[\begin{array}{l} {\bar{p}}_{1}\\ {\bar{p}}_{2} \end{array}\right]$$

It is easy to find that Equation (12) is similar to the form of Equation (2) mentioned above, which indicates that the steady-state position $p^{*}(t)$ of the system can be obtained through the transformation of ξ; that is, the steady-state solution of the system can achieve the expected similar formation of ξ, and the speed of the followers tend to the constant speed $V_{0}$ of the leaders. Equation (13) means that when the leader is traveling at a specified speed, the initial relative positions of the two leaders, ${\bar{p}}_{1}$ and ${\bar{p}}_{2}$, determine the size of the final formation, and that it must be a combination of translation, rotation, and scaling of the base formation ξ.

### 3.3. Global Navigation

In this section, on the basis of the previous section, we consider introducing the multi-robot system that has stably realized dynamic similar formation into the specified map in a two-dimensional plane, in order to realize the global navigation of the multi-robot system.

**Figure 2:**
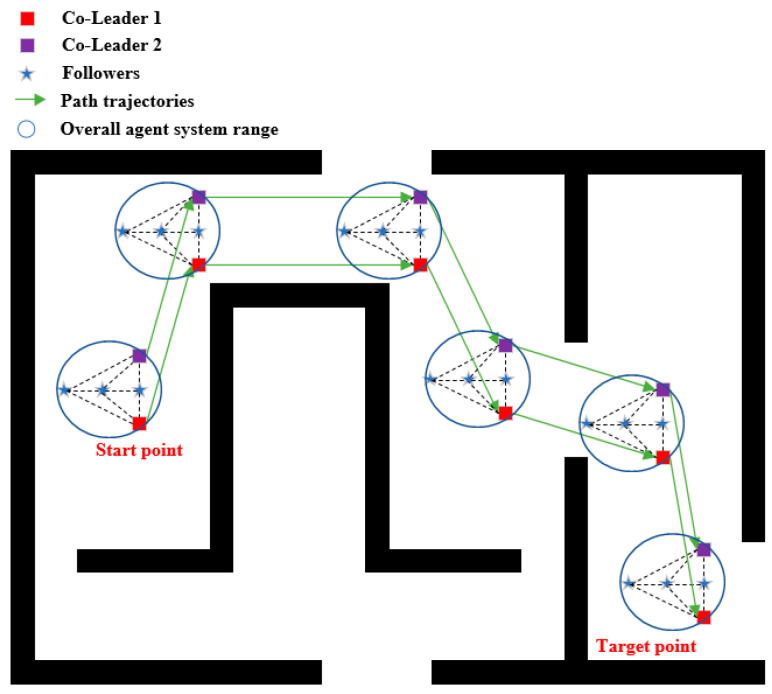
Global navigation schematic of a multi-robot system.

Figure 2 shows the global navigation schematic of the multi-robot system. Firstly, we set the map in a two-dimensional plane coordinate system, set the starting and ending points and obstacles, and then use the path planning algorithm to find the optimal path. It can be observed that we have taken into account the overall radius of the system based on the conventional path navigation algorithm. We treat the entire multi-robot system as a whole circular when considering path planning, ensuring that the individual robots in the system reach the destination in a collision-free manner while maintaining the specified formation.

As verified in the previous section, the control protocols (9) and (10) can make the system maintain similar formation under the motion state, and the system eventually maintains stable convergence. Therefore, it is only necessary to assign the target path to two leaders to realize the global navigation of the whole multi-robot system, and their control protocol becomes as follows:

(14) $$\left\{ \begin{array}{l} {\dot{p}}_{i}=\gamma_{i}\;i=1,2\\ {\dot{p}}_{i}=\gamma_{i}+d_{i}\sum_{j\in N_{i}}w_{ij}(p_{j}-p_{i})\;i=3,4,\cdots ,n \end{array}\right.$$

(15) $$\left\{ \begin{array}{l} \gamma_{i}=f(l_{i})\;i=1,2\\ {\dot{\gamma }}_{i}=\sum_{j\in N_{i}(G_{v})}a_{ij}(\gamma_{j}-\gamma_{i})\;i=3,4,\cdots ,n \end{array}\right.$$

where $l_{i}$ represents the planned target path curve; $f(l_{i})$ represents the corresponding velocity function.

In this section, $l_{i}$ can be obtained using most optimal path algorithms. In order to facilitate subsequent experimental verification, the following path search Algorithm 1 is given in this section:
**Algorithm 1:** Path search algorithm for multi-robot system**Input:** Map boundary and obstacle coordinates, the overall radius of the system R, starting and ending point coordinates **Output:** Planned path node set $P_{a}=\left\{S_{0},S_{1},S_{2}\cdots S_{n}\right\}$ 1 Put the starting point into the buffer set $P_{t}$2 **while** True **do**3   Take the node with the minimum g(n)+h(n) value in $P_{t}$ and put it into set $P_{a}$.4   **if**  The current node is the end point  **then**5      End the loop.6   **end if**7   Iterate through the neighboring nodes of the current node that are not in set $P_{a}$.8   **if**  The neighboring node is in set $P_{t}$ **then**9      Update the g(n) value of the node10   **else**11      Calculate the g(n) value of the neighboring node, and put it into set $P_{t}$
12   **end if**13 **end while**14 **return** $P_{a}$


The algorithm searches for a unique path from the starting point to the ending point by traversing around the nodes and selecting the minimum path cost. This path is stored in $P_{a}$ as a node set, where g(n) is the actual cost from the initial point to the current node n and h(n) is the heuristic function, which is the estimated cost of the best path from the current node n to the target point. The heuristic function h(n) can be set by itself according to specific map scenes and actual needs.

According to the above algorithm, the concrete expression of $f(l_{i})$ can be as follows:

(16) $$\left\{ \begin{array}{l} V_{x}=S_{(T+1)x}-S_{Tx}\\ V_{y}=S_{(T+1)y}-S_{Ty} \end{array}\right..$$

In (16), $V_{x}$ and $V_{y}$ are the fractional velocities of on the horizontal and vertical axes, $S_{Tx}$ and $S_{Ty}$ are the transverse and vertical values of the *T*-th node in the set $P_{a}$, where $T\in {Ν}^{+}$ increases in an integer manner with time t.

### 3.4. Narrow Lane Traffic

**Figure 3:**
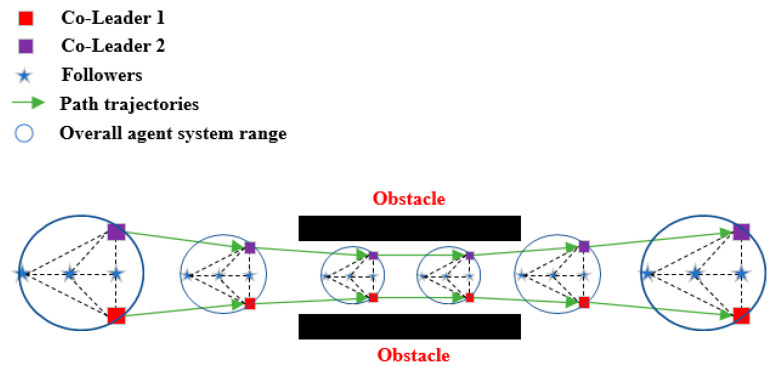
Narrow corridor access schematic of a multi-robot system.

In this section, based on the realization of the global navigation of a multi-robot system in the previous section, we consider increasing the overall system’s processing capacity for special scenes such as the narrow channel, so as to increase the trafficability of the overall navigation of the system. Figure 3 is the schematic diagram of a multi-robot system passing through a narrow channel. On the basis of expecting the normal movement of the whole system formation, the whole system can enter the narrow channel smoothly through the reduction in the formation, and gradually recover the formation after passing.

We add a relative distance-based control strategy to the controller shown in Equation (9). It can be seen from Equation (13) that the desired formation size is related to the distance between the two leaders. Therefore, this control strategy only needs to be applied to the leaders. In order to realize the formation passing of such special map scenes as narrow channels, a novel controller is designed as follows:

(17) $$\left\{ \begin{array}{l} {\dot{p}}_{i}=\gamma_{i}+k(p_{2}-p_{i})({\left\|p_{2}-p_{i}\right\|}^{2}-r^{2})\;i=1\\ {\dot{p}}_{i}=\gamma_{i}+k(p_{1}-p_{i})({\left\|p_{1}-p_{i}\right\|}^{2}-r^{2})\;i=2\\ {\dot{p}}_{i}=\gamma_{i}+d_{i}\sum_{j\in N_{i}}w_{ij}(p_{j}-p_{i})\;i=3,4,\cdots ,n \end{array}\right.$$

where k is the adjustment parameter of formation scaling speed, $\left\|p_{2}-p_{1}\right\|$ and $\left\|p_{1}-p_{2}\right\|$ are the Euclidean distance between the two leaders, and r is the preset expected distance between the leaders. By changing this value, the expected formation size can be adjusted.

In order to meet the actual needs of global navigation, the corresponding speed controller is given in combination with Equations (15) and (16), and its complete expression is as follows:

(18) $$\left\{ \begin{array}{l} \gamma_{i}=(S_{(k+1)x}-S_{kx})V_{x}+(S_{(k+1)y}-S_{ky})V_{y}\;i=1,2\\ {\dot{\gamma }}_{i}=\sum_{j\in N_{i}(G_{v})}a_{ij}(\gamma_{j}-\gamma_{i})\;i=3,4,\cdots ,n \end{array}\right.$$

Similarly, the matrix expression of the system equation of state can be given as follows:

(19) $${\dot{p}}_{l}=\left[\begin{array}{l} \gamma_{1}+k(p_{2}-p_{1})({\left\|p_{2}-p_{1}\right\|}^{2}-r^{2})\\ \gamma_{2}+k(p_{1}-p_{2})({\left\|p_{1}-p_{2}\right\|}^{2}-r^{2}) \end{array}\right],$$

(20) $$\left[\begin{array}{l} {\dot{p}}_{f}\\ {\dot{\gamma }}_{f} \end{array}\right]=Z\left[\begin{array}{c} p_{f}\\ \gamma_{f} \end{array}\right]+X\left[\begin{array}{c} p_{l}\\ \gamma_{l}1_{2} \end{array}\right].$$

where, $p_{l}={\left[p_{1},p_{2}\right]}^{T}$, $p_{f}={\left[p_{3},p_{4},\cdots ,p_{n}\right]}^{T}$, $\gamma_{l}={\left[\gamma_{1},\gamma_{2}\right]}^{T}$, and $\gamma_{f}={\left[\gamma_{3},\gamma_{4},\cdots ,\gamma_{n}\right]}^{T}$. Meanwhile, let $D_{f}={\left[d_{3},d_{4},\cdots ,d_{n}\right]}^{T}$, and the specific form of Z and X is given as follows:

(21) $$Z=\left[\begin{array}{cc} -D_{f}L_{ff} & I_{n-2}\\ 0 & -H_{ff} \end{array}\right],X=\left[\begin{array}{cc} -D_{f}L_{lf} & 0\\ 0 & -H_{lf} \end{array}\right],$$

Obviously, when Lemma 1 is satisfied and the initial position of the two leaders ${\bar{p}}_{1}\neq {\bar{p}}_{2}$, the steady-state solution of the system has the following form:

(22) $$\left\{ \begin{array}{l} p^{*}(t)=(c_{1}+V_{0}t)1_{n}+e^{\theta j}\left[\frac{r}{\xi_{12}}\right]\xi \\ \gamma^{*}(t)=V_{0}1_{n} \end{array}\right.,$$

where $\xi_{12}=\left\|\xi_{1}-\xi_{2}\right\|$, which represents the relative distance between the leaders in base formation ξ, $c_{1}$, and θ depend on the initial positions of the two leaders and represent the translation and rotation degree of the formation compared to the base formation ξ.

Similar to Equation (12), it is not difficult to see that a steady-state solution of the system satisfies the form described in Equation (2). Therefore, in this control protocol, the multi-robot system can asymptotically and stably form a moving formation with the basic formation shape ξ and the size of the formation depends on the desired distance r between the leaders.

According to the above introduction, we obtain a control protocol that can stably realize the whole formation scaling of the system. Next, Algorithm 2 is given to make the whole system pass through the narrow channel and apply it to the global navigation of the system.
**Algorithm 2:** Global navigation algorithm**Input:** The overall radius of the system R, the expected distance between leaders r, the minimum value of the overall circle $R_{\min}$
**Output:** The state trajectory diagram of the p-value of each agent1 **while** Not at the end **do**2   **if** A narrow channel is detected then3      Let $r=R_{\min}$, the leaders continue to travel at velocity $\gamma_{i}$, the followers are controlled by the controller to follow4   **else**5      Let $r=R_{\min}$, the leader continue to travel at velocity $\gamma_{i}$, the follower is controlled by the controller to follow6   **end if**7 **end while**8 Output the final trajectory diagram

It is assumed that each individual mobile robot carries an on-board sensor to assist in building a map scene and measuring the relative position and velocity of other individual robots. Also, the sensors should have certain recognition and detection abilities for such special scenes as narrow channels. 

When the system moves in the desired formation, once both leaders detect a narrow passage at the front that is smaller than the overall circular diameter of the system, the overall formation is reduced by decreasing the desired distance r between the leaders, which in turn makes the relative distance between the two navigators smaller, so as to enter the passage smoothly. When the passage is successfully passed, the original formation is restored.

In order to enable the above method to be applied in the global navigation of the system, we set an overall circle minimum value $R_{\min}$ for the whole system in advance, which is the minimum value of the aisle that the whole system can pass through. Then, we plan the global path of the system in the target map based on this minimum value of the whole circle diameter $R_{\min}$. When the multi-robot system is marching in the map with the target formation, once it detects a narrow channel, the system formation will be reduced to the minimum value of the overall circle to smoothly pass through the channel. After passing, the original size of the formation should be restored and continue to move to the end point along the planned path.

## 4. Simulation Experiment

### 4.1. Similar Formation

In this section, we consider using a five-robot system and a six-robot system, as shown in Figure 1, to conduct simulation experiments. According to Definitions 1 and 2, the communication topology of the two systems is two-rooted, which provides preconditions for similar formation control. In both systems, individual robot 1 and individual robot 2 are co-leaders, whereas the rest are followers. The formation adopted is the similar formation of ξ as shown in Figure 1. Therefore, relevant parameter settings are given as follows:

$$L_{1}=\left[\begin{array}{ccccc} 0 & 0 & 0 & 0 & 0\\ 0 & 0 & 0 & 0 & 0\\ 1 & 1 & -2 & 0 & 0\\ 0 & 0 & -j & 2j & -j\\ -1+2j & -1-2j & 0 & 0 & 2 \end{array}\right]\;H_{1}=\left[\begin{array}{ccccc} 0 & 0 & 0 & 0 & 0\\ 0 & 0 & 0 & 0 & 0\\ 0 & -1 & 2 & -1 & 0\\ -1 & -1 & 0 & 2 & 0\\ -1 & 0 & 0 & -1 & 2 \end{array}\right]\;D_{1}=diag\left\{\begin{array}{ccccc} 1 & 1 & -2 & -0.03-0.3j & 0.2+0.05j \end{array}\right\}\;L_{2}=\left[\begin{array}{cccccc} 0 & 0 & 0 & 0 & 0 & 0\\ 0 & 0 & 0 & 0 & 0 & 0\\ 0 & -4j & 4+4j & -4 & 0 & 0\\ 0 & 0 & 1.2-1.6j & -0.2+3.6j & -1-2j & 0\\ -2j & 0 & 0 & 1+2j & -1.8-0.4j & 0.8+0.4j\\ 0 & -2-1j & 0 & 0 & 1-2j & 1+3j \end{array}\right]\;H_{2}=\left[\begin{array}{cccccc} 0 & 0 & 0 & 0 & 0 & 0\\ 0 & 0 & 0 & 0 & 0 & 0\\ 0 & -1 & 2 & -1 & 0 & 0\\ 0 & 0 & -1 & 2 & -1 & 0\\ -1 & 0 & 0 & -1 & 3 & -1\\ 0 & -1 & 0 & 0 & -1 & 2 \end{array}\right]\;D_{2}=diag\left\{\begin{array}{cccccc} 1 & 1 & -4j & 2 & -2j & 1 \end{array}\right\}$$

where $L_{1}$, $H_{1}$, and $D_{1}$ are five-robot system formation parameters, and $L_{2}$, $H_{2}$, and $D_{2}$ are six-robot system formation parameters.

It can be found that for the diagonal matrices $D_{1}$, $D_{2}$ selected for two multi-robot systems, all the eigenvalues of −DL corresponding to the system can be located in the left half plane of the coordinate axis. Meanwhile, let the two co-leaders move with a common velocity:

(23) $$v_{i}=\left\{ \begin{array}{l} x=kt\\ y=ktj \end{array}\right.(i=1,2),$$

In the five-robot system $v_{1}$ is taken as k=0.5, and in the six-robot system $v_{2}$ is taken as k=25, and the initial positions of the robotic individuals in the two systems can be as follows:

$$\left\{ \begin{array}{l} P_{1}=\left[\begin{array}{ccccc} 0+1j & 0+2.3j & -2.1-2.1j & -2.2+1.9j & 3-1.2j \end{array}\right]\\ P_{2}=\left[\begin{array}{cccccc} 1+2j & 1+3.3j & 2-2j & -1+3j & 3-3j & 2+3j \end{array}\right] \end{array}\right.$$

**Figure 4:**
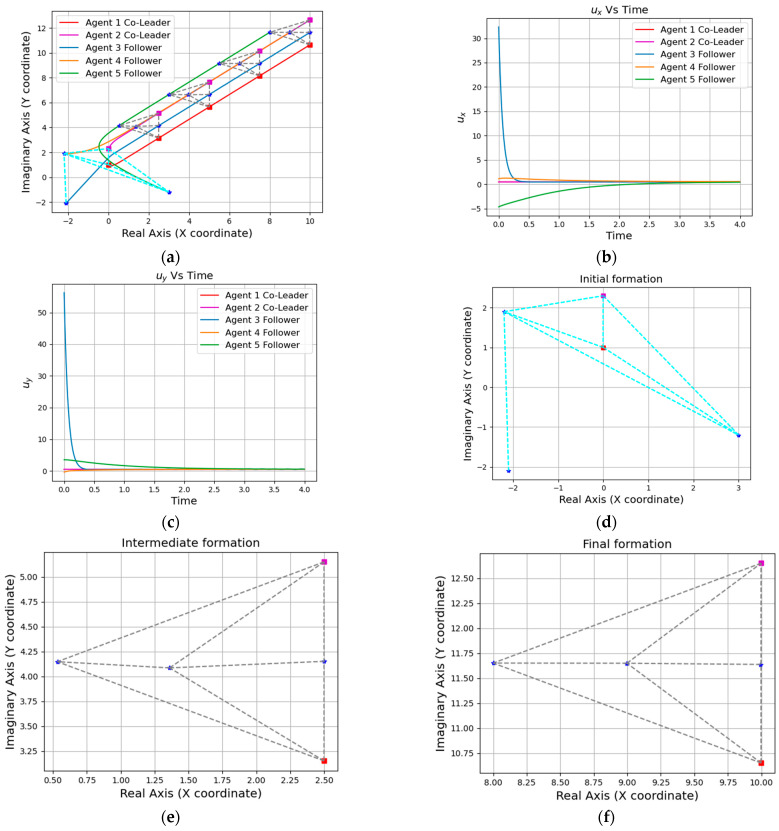
A formation of the five-agent system: (**a**) Position trajectories of agents. (**b**) Evolution of formation errors (ux). (**c**) Evolution of formation errors (uy). (**d**) Initial formation. (**e**) Intermediate formation. (**f**) Final formation.

**Figure 5:**
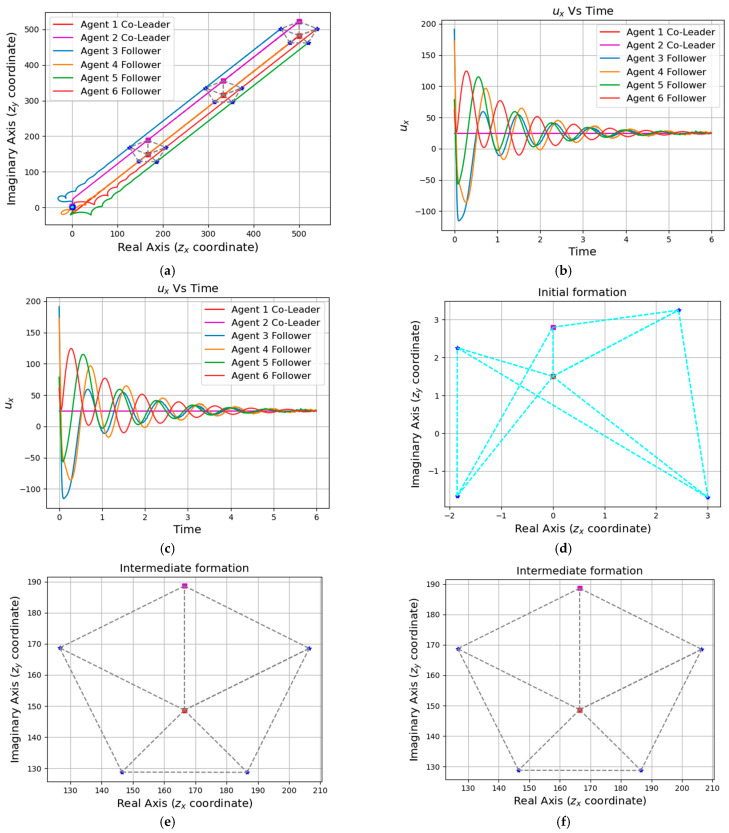
A formation of the six-agent system: (**a**) Position trajectories of agents. (**b**) Evolution of formation errors (ux). (**c**) Evolution of formation errors (uy). (**d**) Initial formation. (**e**) Intermediate formation. (**f**) Final formation.

**Figure 6:**
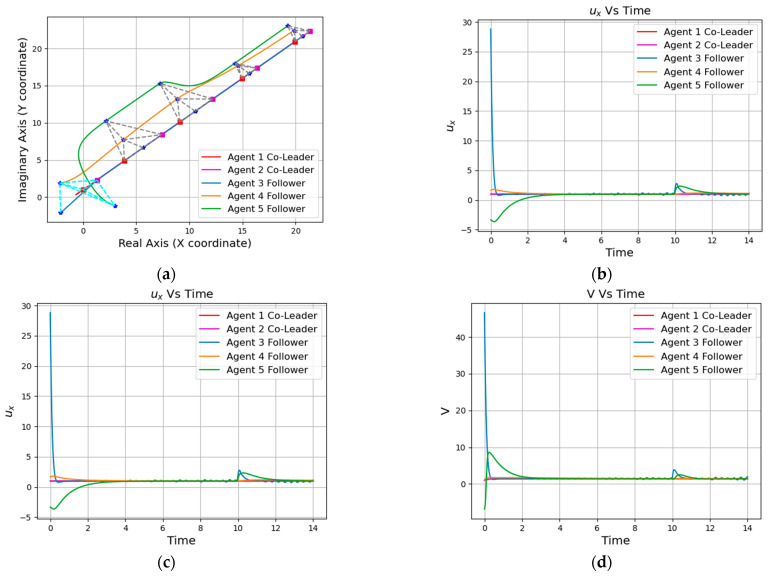
Similar formation of the five-agent system: (**a**) Position trajectories of agents. (**b**) Evolution of formation errors (ux). (**c**) Evolution of formation errors (uy). (**d**) Evolution of formation errors (V).

**Figure 7:**
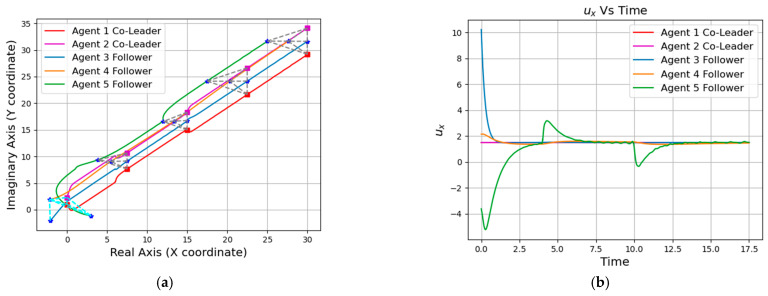

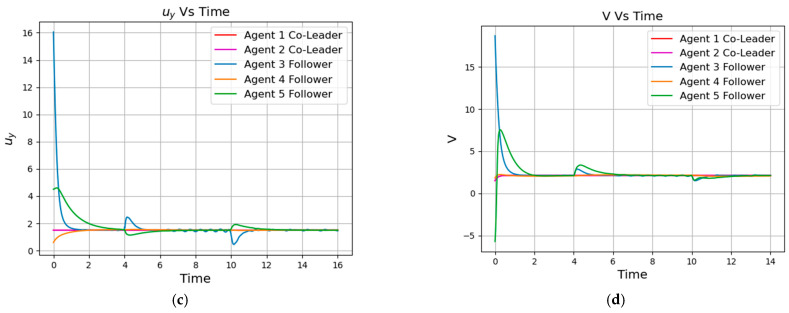
Scalable formation of the five-agent system: (**a**) Position trajectories of agents. (**b**) Evolution of formation errors (ux). (**c**) Evolution of formation errors (uy). (**d**) Evolution of formation errors (V).

Simulation results are shown in Figure 4, Figure 5, Figure 6 and Figure 7. Among them, Figure 4 and Figure 5a show the motion trajectory diagram of the five-robot system and six-robot system, respectively, indicating that both systems can realize the formation, as shown in Figure 1, and move at the specified speed. Figure 4 and Figure 5b,c depict the curve of the errors of control input and the expected state input of each individual robot in the two systems over time, where (b) is the error on the real number line, and (c) is the error on the complex number line. Figure 4 and Figure 5d–f, respectively, show the initial, intermediate, and final formations of the two systems.

Next, we take the five-robot system as an example to simulate and verify the similar formation functions of the system. It can be seen from the position trajectory diagram in Figure 6a that the five-robot system realized the rotation and reduction of the formation. In Figure 6b,c, the fluctuations of the five-robot system at 10 s also verified the change in formation scaling. In this paper, the multi-robot system is required to realize formation passing on a narrow channel and to recover the formation after passing, so it is necessary to realize the secondary scaling of the formation. The position trajectory diagram in Figure 7a shows the reduction in the formation of the five-robot system and the implementation of the restoration function. At the same time, the two fluctuations of the control input waveform in Figure 7b,c also verified the change in the formation reduction and recovery.

### 4.2. Simulation of Global Path Navigation

**Figure 8:**
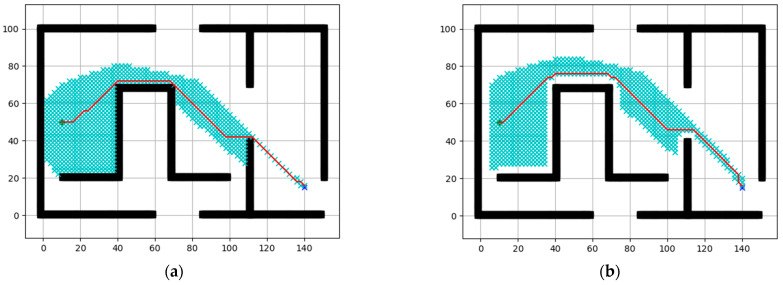
Path planning: (**a**) Initial path planning. (**b**) Path planning considering the overall radius of agents (R = 5).

In this section, we conducted simulation verification of formation navigation for two multi-robot systems. Firstly, the simulation implementation of the path planning Algorithm 1 is carried out. The simulation results are shown in Figure 8, where the green dot represents the starting point, the blue dot represents the destination, the black blocks represent obstacles, the cyan area represents the expanded neighborhood during path search, and the red line segment represents the final path. Next, to fulfill the overall formation navigation of the multi-robot system, we introduced the consideration of the overall radius R of the system. This ensures that the planned paths always maintain an appropriate distance from the map boundaries and obstacles. The specific simulation results are shown in Figure 8b.

**Figure 9:**
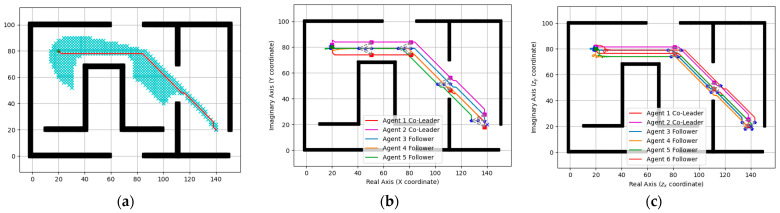
Global path planning (R = 8): (**a**) Initial path. (**b**) Path navigation for the five-agent system. (**c**) Path navigation for the six-agent system.

**Figure 10:**
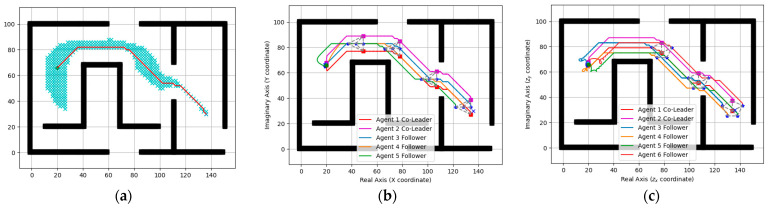
Global path planning (R = 12): (**a**) Initial path. (**b**) Path navigation for the five-agent system. (**c**) Path navigation for the six-agent system.

Then, we simulate the path navigation algorithm under different R values (the specific value of R is positively correlated with the actual formation size of the multi-agent system) and make two multi-robot systems move along the planned path. As can be seen from Figure 9 and Figure 10, both of the two robot systems can reach the destination smoothly without collision.

### 4.3. Simulation of through a Narrow Corridor

**Figure 11:**
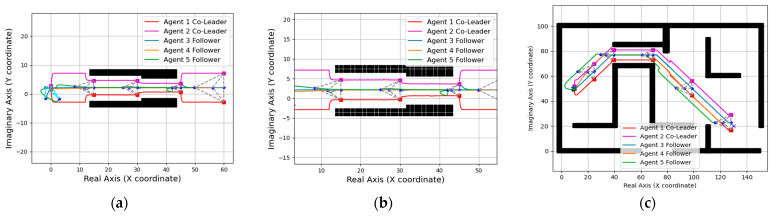
(**a**) Simulation of the five-agent system passing through a narrow corridor. (**b**) Detailed drawing of a narrow corridor. (**c**) Path navigation for the five-agent system that includes passing narrow corridors.

**Figure 12:**
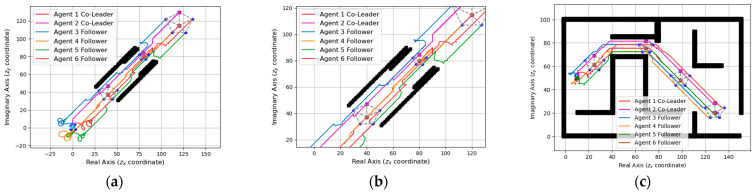
(**a**) Simulation of the six-agent system passing through a narrow corridor. (**b**) Detailed drawing of a narrow corridor. (**c**) Path navigation for the six-agent system that includes passing narrow corridors.

In this section, we simulate a special case of two multi-robot systems passing through a narrow corridor of different widths in a suitable formation size. Initially, the systems move forward along the specified route with the initial formation size. When the co-leader detects that the map obstacle wall ahead is too close, the entire system undergoes a reduction in the formation size. Once the passage is passed, the system resumes its original formation. The simulation results are shown in Figure 11 and Figure 12a. Furthermore, we integrated the path navigation of the two systems into the simulation. From Figure 11 and Figure 12b, it can be observed that both the multi-robot systems can successfully navigate through narrow corridors in the path navigation and reach the specified destination.

## 5. Conclusions

This paper studies a navigation control problem of multi-robot systems in the leader–follower network. A distributed linear control strategy for multiple robots based on a complex Laplacian matrix is proposed to drive a group of robot agents to realize the dynamic moving similar formation of the system as a whole. Then, the path navigation algorithm is combined with the dynamic moving similar formation control to realize the global formation navigation of the whole system, and through a controller that can scale the formation size, the accessibility of the whole system to special scenes like long and narrow corridors is increased. The simulation results show the effectiveness of the proposed method. Further work will continue to address the problem of collision with dynamic obstacles, and we plan to apply the theoretical research results in practice to engineering applications.

## Data Availability

Data are contained within the article.
